# Gingival Enlargement During Orthodontic Treatment: A Narrative Review

**DOI:** 10.7759/cureus.111127

**Published:** 2026-06-19

**Authors:** Amalia Syrioti, Christina Charisi, Vasileios Zisis, Nikolaos Spantidakis, Filippos Fytros, Konstantinos Poulopoulos, Thomas Chontos, Petros Papadopoulos, Stefanos Zisis, Nikolaos Shinas, Athanasios Poulopoulos, Smaragda Diamanti

**Affiliations:** 1 Oral Medicine and Pathology, European University Cyprus, Nicosia, CYP; 2 Oral Medicine and Pathology, Aristotle University of Thessaloniki, Thessaloniki, GRC; 3 Pediatric Dentistry, Aristotle University of Thessaloniki, Thessaloniki, GRC; 4 Dentoalveolar Surgery, Aristotle University of Thessaloniki, Thessaloniki, GRC; 5 Implantology and Oral Radiology, Aristotle University of Thessaloniki, Thessaloniki, GRC; 6 First University Clinic of Ophthalmology, Athens General Hospital (GNA) “Georgios Gennimatas”, Athens, GRC; 7 Periodontology, Rheinisch-Westfälische Technische Hochschule (RWTH) Aachen University, Aachen, DEU; 8 Oral and Maxillofacial Radiology, Boston University Henry M. Goldman School of Dental Medicine, Boston, USA

**Keywords:** gingival enlargement, gingival hyperplasia, gingival overgrowth, orthodontic appliances, orthodontic treatment, periodontal changes

## Abstract

Gingival enlargement is commonly seen during orthodontic treatment, specifically in individuals with fixed appliances. Its clinical significance stems from its multifactorial etiology. The purpose of this narrative review is to evaluate the available evidence related to the causes, influential components, symptomatic profiles, and therapeutic approaches to gingival enlargement during orthodontic treatment. An electronic literature research was carried out in PubMed without any limitations. A total of 10 studies were chosen, incorporating one randomized clinical trial and nine observational studies, including cross-sectional descriptive, histological, clinical observational, clinical and histological, and case report studies. The selected articles were focused on gingival alterations in individuals with either fixed or removable orthodontic devices. Then, the determinations were synthesized narratively and thematically. The outcomes propose that multiple factors are associated with gingival enlargement. Fixed orthodontic appliances are associated with an increased risk of gingival enlargement and may influence immune-mediated responses, resulting in thickened epithelium and proliferation of connective tissue, as demonstrated by histological findings. The overall therapy time, the type of device, and local irritants are considered influential factors. The treatment can be either conservative, such as improved dental health and the administration of antimicrobials, or invasive, including gingival excision or laser surgery. Laser therapy may be considered less invasive due to minimal hemorrhage and better recovery outcomes, although based on limited evidence. Gingival enlargement associated with orthodontic devices is a condition influenced by multiple factors, including microbial, mechanical, and substance-related factors. Even though inflammation can be reduced with minimally invasive treatments, surgical interventions are often essential in ongoing cases. Additional longitudinal research is essential to explain the etiology and strengthen prophylactic and healing approaches. The objective of this narrative review was to evaluate existing information on gingival enlargement associated with orthodontic appliances, with a particular focus on the underlying mechanisms, clinical consequences, and therapeutic strategies.

## Introduction and background

Gingival enlargement is a disorder that can develop due to idiopathic, pharmaceutical, or immunological factors and is distinguished by significant overgrowth in the dimension of the gingiva [1,2]. Increased gingival diameter modifies gingival structure, and sometimes functional and esthetic damage represent the clinical manifestations [2,3]​. Plaque formation and persistent inflammatory mechanisms are frequently associated with the disorder and play a crucial role in its etiology [4]. Gingival enlargement may result in localized pain, compromise proper oral hygiene practices, and place individuals at risk for further periodontal issues if interventions do not occur [3]. Even though orthodontic therapy is often utilized to enhance function and correct dental malocclusion, it has been correlated to a range of periodontal complications [5,6]. Brackets, bands, and archwires are some of the fixed orthodontic devices that result in plaque-prone sites that impair oral hygiene practices and promote bacterial growth [7,8]. When adequate plaque regulation fails to be maintained, this alteration in the oral cavity can culminate in greater levels of biofilm buildup and subsequently gingival inflammation [4,8-10]. Orthodontic therapy is frequently associated with alterations to periodontal parameters, like elevated plaque index, gingival index, and bleeding on probing, as reported in clinical and microbiological analyses [9,11,12]. These modifications indicate how susceptible patients receiving orthodontic treatment are to inflammatory gingival diseases. Microbial, mechanical, and host-induced determinants are combined and associated with gingival enlargement, which is generally considered a multifactorial disorder [13,14]. Excessive plaque formation amplifies and produces gingival expansion, while the installation of orthodontic appliances is associated with prolonged mechanical influence on gingival tissues [8,13]. Gingival enlargement also progresses from disturbances in the oral microbiota and the immune system response, with inflammatory mediators representing an essential component of tissue growth. It has been suggested that certain substances in orthodontic equipment, such as metal alloys, could affect cellular responses and induce changes in the gingiva [14]. Gingival enlargement throughout orthodontic procedures can be impacted by a variety of parameters, such as oral hygiene, the therapy period, and patient adherence [15]. Enhanced dental hygiene habits and periodontal care are frequently capable of managing the condition, but in severe or recurrent cases, surgical treatment is required to reconstruct normal gingival anatomy [16]. To accomplish optimal therapeutic outcomes and prevent recurrence, prompt detection and a comprehensive approach combining orthodontic and periodontal procedures are vital. Understanding the origins, risk factors, and treatment of gingival enlargement is of paramount importance, given the increasing number of individuals undergoing orthodontic therapy and the clinical value of this condition.

## Review

Material and method

A comprehensive electronic literature search was performed on PubMed to find pertinent research studies assessing the connection between orthodontic procedures and gingival enlargement. Prior to the research, the eligibility requirements had been established. The studies that were reviewed examined gingival alterations or changes in the gingiva related to orthodontic devices. All studies were viewed as eligible with no limitation on year of publication, even though only published studies in English were used. In the analysis, narrative reviews, methodological papers, and studies that were partially or not related to orthodontically triggered gingival enlargements were excluded. The search was done using the keywords “gingival hyperplasia AND orthodontic appliances” on March 2. The method of approach selection criteria led to the detection of 41 articles. With the observation of the titles and abstracts, 20 publications were eliminated due to partial relation to the topic, three were completely irrelevant, and eight were excluded due to the unavailability of their abstracts for evaluation.

**Figure 1:**
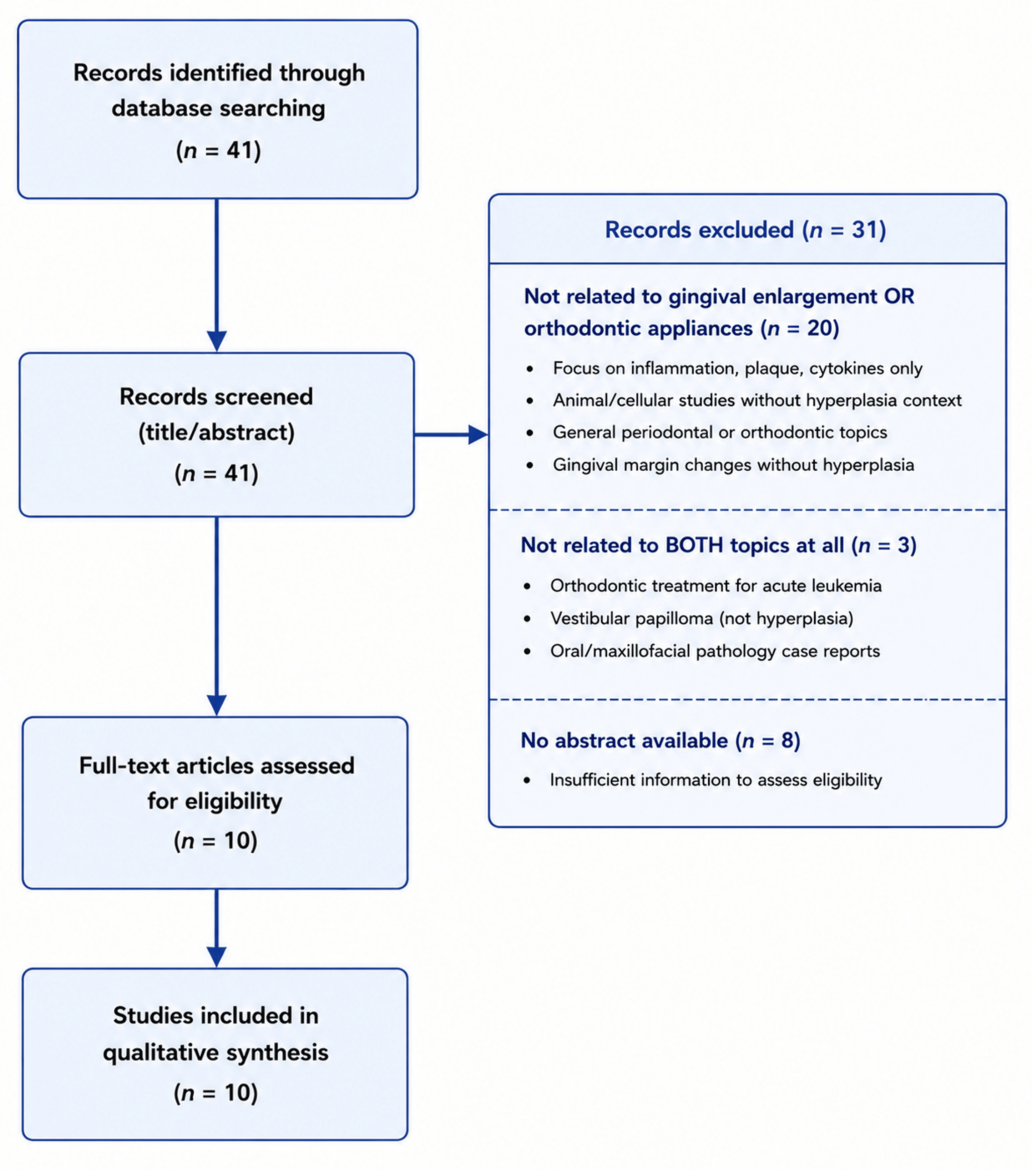
Flowchart illustrating the study selection process, including article identification, screening, eligibility, and inclusion. Our study constitutes a narrative review with a structured literature search.

The 20 partially related publications were eliminated (n=20) since they did not meet the main objective of this review. Particularly, the following considerations led to the classification of studies as partially related. Gingival overgrowth was not monitored as the main outcome (n=8), but gingival inflammation or other periodontal alterations were investigated in orthodontic patients. Orthodontic appliances were present without any evaluated association with gingival overgrowth (n=5), meaning no causal or correlational link between appliances and tissue enlargement was assessed. The study did not directly measure tissue enlargement (n=4) but instead focused on plaque accumulation, gingival indices, or microbial alterations as primary outcomes. Gingival overgrowth was only reported as a secondary or incidental finding (n=2), without systematic measurement or validated diagnostic criteria. Gingival alterations were not the primary aim of the study (n=1), as the focus was on allergic responses, hypersensitivity reactions, or effects related to nickel exposure rather than clinically defined gingival overgrowth. A total of 10 studies were included that met the selection criteria. Of those, one was a randomized clinical trial, five were observational studies (including cross-sectional, case-control, and histological studies), and four were case reports (Figure 1).

Results

A total of 10 studies were extracted for further analysis. The main reviewers were A.S., C.C., N.S., and F.F., under the supervision of V.Z. The main features and important outcomes of the selected studies are presented in Tables 1-3.

**Table 1:** Appliance type, treatment duration, and the role of plaque accumulation (n=4).

S. no.	Studies	Study design	Sample	Type of appliance	Key findings
1.	Vincent-Bugnas et al. (2021) [17]​	Cross-sectional descriptive study	193 orthodontic patients, ages 9-30 years old	Fixed orthodontic appliances (metal, self-ligating, ceramic brackets)	Gingival enlargement was observed in almost half of the patients; metal brackets and duration of treatment were associated with increased risk of gingival enlargement; the amount of plaque was not directly associated. The prioritization of predisposing factors of gingival hyperplasia during orthodontic treatment is not always straightforward. Other factors may be at play mediating this condition, such as the mechanical irritation of braces, the presence of excess cement, hormonal factors, and hypersensitivity to materials
2.	Simon et al. (2020) [18]​	Clinical histological study	44 patients (22 male and 22 female)	Fixed arch wires (AWD) and vacuum-formed retainers (VFR)	Fixed orthodontic appliances were associated with increased risk of gingival enlargement, increased gingival stress, and inflammation. VFR had the smallest effects; men presented slightly increased stress
3.	Rădeanu et al. (2024) [19]​	Clinical observational study	Out of 327 patients, 74 were selected who had gingival enlargement related to orthodontic treatment (age range: 14-56 years)	Fixed orthodontic appliances	Fixed orthodontic appliances were associated with increased risk of gingival enlargement and inflammation; this condition was also associated with plaque, calculus, and local irritation
4.	Jadhav et al. (2013) [20]​	Case report	A 19-year-old male orthodontic patient	Fixed orthodontic appliances	Chronic inflammatory gingival inflammation, together with the accumulation of plaque during orthodontic treatment, was associated with gingival enlargement and was managed with changes to improve oral hygiene, together with periodontal therapy

**Table 2:** Orthodontic-appliance and material-related factors in the included studies (n=3).

S. no.	Studies	Study design	Sample	Type of appliance	Key findings
1.	Gursoy et al. (2007) [21]​	Clinical and histological study	10 orthodontic patients (8 females and 2 males), mean age: 15.4 years, with paired hyperplastic and healthy gingival samples	Fixed orthodontic appliances	Nickel ion release has been proposed as a possible contributory factor influencing epithelial proliferation and inflammatory responses, which are associated with gingival enlargement
2.	Bellamine et al. (2012) [22]​	Case report	A 13-year-old orthodontic patient	Fixed orthodontic appliance	Orthodontic treatment was associated with increased risk of gingival enlargement, managed with an apically positioned flap and enhanced plaque control to approve continuation of the orthodontic treatment
3.	Barack et al. (1985) [23]​	Case report	1 orthodontic patient	Fixed orthodontic appliance	Orthodontic treatment was associated with localized periodontal issues, such as gingival enlargement, which were resolved by improved oral hygiene and periodontal care

**Table 3:** Patient-related factors and therapeutic strategies in the included studies (n=3). GI: gingival index; PI: plaque index

S. no.	Studies	Study design	Sample	Type of appliance	Key findings
1.	Farhadian et al. (2015) [24]​	Randomized clinical trial	72 orthodontic patients with a minimum of 2 sites of gingival inflammation (separated into 4 groups)	Fixed orthodontic appliances	The 4 groups were as follows: manual toothbrush, electric toothbrush, manual Persica, and chlorhexidine. The group that used chlorhexidine had reduction in gingival index and hyperplastic index; Persica had same outcome; the electric toothbrush improved GI/PI compared to using only manual, but none of them fully reduced gingival enlargement
2.	Convissar et al. (1996) [25]​	Case report	1 orthodontic patient	Fixed orthodontic appliance	Gingivectomy by lasers was associated with reduction of gingival enlargement associated with orthodontic appliances, helping orthodontic treatment to continue
3.	Gama et al. (2007) [26]	Clinical interventional study	10 orthodontic patients (75 anterior teeth with gingival hyperplasia)	Fixed orthodontic appliances	Gingivectomy with CO_2_ laser improved crown length and decreased gingival sulcus depth, being associated with effective treatment of gingival enlargement associated with orthodontic appliances

In all the reviewed studies, gingival enlargements were frequently connected with fixed orthodontic appliances [17-20]. As opposed to alternative orthodontic treatment methods, all research concluded that fixed appliances were linked with a greater likelihood of gingival enlargement, though the extent and contributing factors differed between publications.

Bracket type and therapy length are indications of appliance-associated parameters that are regularly observed. In a particular investigation, metal brackets and prolonged orthodontic use were correlated with a greater likelihood of gingival enlargement [17]. Further histological findings demonstrated that while vacuum-formed retainers exhibited little gingival alteration, fixed orthodontic appliances were related to increased gingival stress, connective tissue proliferation, and epithelial thickening [18].

Studies additionally demonstrated local oral causes and inflammation. In individuals undergoing fixed orthodontic treatment, gingival enlargement was associated with plaque buildup, calculus formation, and local irritants such as poorly placed restorations and cavities [19]. However, research revealed no relationship between the degree of gingival enlargement and plaque concentration [17]. Therefore, plaque may be a qualitative rather than a quantitative factor. Plaque may act as a precursor, but thereafter its quantity may play a secondary role. Furthermore, a case report described the use of periodontal therapy and proper oral hygiene to treat chronic inflammatory gingival overgrowth caused by plaque accumulation during orthodontic treatment [20].

In general, gingival enlargement was associated with fixed orthodontic appliances across all studies, despite differences in determinants such as appliance characteristics and oral hygiene practices [17-20]. Across the included investigations, gingival enlargement was attributed to orthodontic appliances and correlated with material exposure, with all studies involving fixed orthodontic devices [21-23].

A clinical and histopathological examination assessing nickel ion exposure in gingival tissues evaluated material-associated indices. Both enlarged and healthy gingival samples had similar nickel concentrations; however, low-level exposure was associated with epithelial growth, whereas higher levels were associated with cytotoxic effects [21].

Gingival enlargement during fixed orthodontic treatment was also documented in case studies. In one instance, gingival enlargement during orthodontic therapy was managed through the use of an apically positioned flap, combined with enhanced plaque control, enabling treatment to proceed [22]. Localized gingival overgrowth associated with prolonged orthodontic therapy was noted in another case report [23]. This condition disappeared with proper oral sanitation and periodontal care. In all included papers, gingival enlargement was demonstrated to be associated with fixed orthodontic devices. Additional research indicated a relationship among material exposure, plaque control, and alterations in periodontal tissue [21-23].

Chemical plaque control, mechanical oral hygiene measures, and surgical techniques were utilized to treat gingival enlargement during orthodontic treatment [24-26]. Individuals undergoing fixed orthodontic treatment with concurrent gingival enlargement were managed using these approaches. Chlorhexidine, Persica mouthwash, electric toothbrush use, and manual brushing were among oral hygiene approaches that were used in a randomized clinical trial [24]. Gingival and plaque concentrations were reduced across all intervention groups, even though gingival enlargement was not completely eliminated by any intervention. Compared to manual brushing alone, the incorporation of chemical agents and electric toothbrushes showed greater improvements in gingival and plaque scores [24].

Furthermore, there were additional studies on surgical management methods. In orthodontic clients, CO_2_ laser gingivectomy was responsible for improvements in clinical parameters such as crown length and gingival sulcus depth, along with a decrease in gingival enlargement [26]. In the same way, excess gingival tissue was removed via laser-assisted gingivectomy, which enabled the continuation of orthodontic therapy [25]. Overall, non-surgical dental care methods and laser-assisted surgical interventions were employed to control gingival enlargement during orthodontic treatment. After the completion of the therapy, gingival inflammation and tissue overgrowth appeared to be reduced [24-26].

Discussion

A comprehensive overview of gingival enlargement associated with orthodontic treatment can be obtained from the 10 cited studies, which include observational studies, cross-sectional studies, clinical trials, and case reports. The main concept is that gingival enlargement is a clinically relevant result of orthodontic appliances. This is confirmed by consistent evidence that can be seen in each paper regardless of differences in study design, sample size, or main findings [18-20,24]. Evaluating these analyses also indicates major differences in the accuracy of the findings, with smaller studies providing more clinical knowledge and larger studies offering statistical relevance.

A distinction is evident among investigations related to cases compared to studies with substantial sampling. As an example, due to greater population numbers, researchers such as Rădeanu et al. and Farhadian et al.​ present stronger information regarding the occurrence-related risk determinants, facilitating more accurate application of the outcomes [19-24]. On the other hand, even with a limited number of samples, case studies like Jadhav et al. [20], Bellamine et al. [22], and Barack et al. present significant findings on clinical manifestations, the condition’s evolution, and treatment approaches [23]. This contrast demonstrates that larger studies identify the general incidence and risk characteristics, while small-scale studies play a major role in evaluating different responses, and the coherence of conclusions from the different kinds of studies increases the research’s total reliability.

Considering the pathogenesis, the majority of the investigations seem to agree that plaque buildup is a key component in the progression of gingival enlargement throughout orthodontic therapy [18-20,22]​. Retentive zones resulting from fixed orthodontic appliances prevent adequate dental maintenance, leading to tissue enlargement, plaque accumulation, and gingival irritation. A closer look at the information, however, reveals that gingival enlargement is not entirely caused by plaque. Studies such as those by Gursoy et al. and Farhadian et al. present instances where enlargement was detected despite good oral health, demonstrating that other variables might be relevant [21,24]. Furthermore, Rădeanu et al.​ discovered calculus formation, caries, and inadequately fitted restorations as significant local irritants [19].

Furthermore, rather than only growing the concentration of plaque, a recent study demonstrated that orthodontic appliances might trigger microbial dysbiosis [27]. Gingival enlargement might be worsened by alterations in the subgingival microbiome, with a tendency toward pathogenic species [5,7]. Al-Mutairi et al., who found significant microbial shifts in the periodontal microbiota associated with orthodontic appliances, confirmed this [27]. Hence, the outcomes indicate a multifactorial etiology that contains mechanical, microbiological, and host-related factors.

Another field where the research coincides is the significance of inflammation and host response. Gingival enlargement could refer to an inflammatory reaction that extends from an initial immune response to a greater proliferative state, as shown by histological and clinical findings [17,20]​. Since equivalent plaque amounts may result in different clinical manifestations, this further underscores the importance of individual heterogeneity in host responses. Moreover, as Teodorescu et al.​ emphasize, systemic and patient-associated factors might influence periodontal health during orthodontic therapy [28]. Gursoy et al.​ broaden the etiological paradigm by highlighting the probable involvement of material-associated components that may stimulate epithelial expansion, such as the release of metal ions from orthodontic devices [21]. In conjunction with the above findings, these observations present a deeper understanding of the fundamental mechanisms triggering gingival enlargement. The role of nickel hypersensitivity continues to be unknown, though. However, there is a hypothesis of nickel ion release from orthodontic devices; specifically, an analysis by Stoyanova-Ivanova et al. demonstrates that hypersensitivity reactions are quite uncommon and cannot serve as a major contributing factor [29]. Amato et al.​ observed that responses to hypersensitivity are individual-specific and not always associated with gingival enlargement, providing further evidence [30].

While analyzing the corresponding studies, the duration of therapy also stands as a significant aspect. Long-term orthodontic treatment is associated with an elevated probability of gingival enlargement [20,23,24,26]. But since various investigations have distinct follow-up intervals, it is hard to establish a specific onset underlying the need for long-term research. The included research studies collectively reveal that the severity of the disease strongly influences therapeutic outcomes. Mild cases are usually treated with proper oral hygiene and periodontal therapy [20,22,23]. The study by Bellamine et al.​ highlights the significance of early and efficient management, presenting that after surgical periodontal procedures, orthodontic treatment can be normally continued ​[22]. Combining literature that examines surgical methods, including laser therapy, could provide better clinical outcomes. According to studies by Convissar et al.​ and Gama et al., CO₂ laser gingivectomy is a favorable therapeutic procedure for gingival enlargement triggered by orthodontics [25,26]. But these results are based on small sample sizes, and there is little evidence to support the claim that they are more effective than traditional techniques.

Periodontal effects might also be influenced by the kind of orthodontic appliance used. Although the data remain contradictory, some studies indicate that self-ligating brackets are associated with reduced plaque accumulation and gingival irritation compared with conventional brackets [31]. On the other hand, by improving oral health and reducing plaque concentration, removable clear aligners may have fewer periodontal effects and possibly fewer chances of gingival enlargement [32]​.

Another crucial consideration is whether gingival enlargement predicts future periodontal disease. Despite the fact that it is usually considered a transient inflammatory condition, enlargement might increase the chances of prolonged periodontal issues [11]​. The discrepancy in study design and outcome evaluation is another significant result from the assessment of the publications. The focus of the featured literature ranges widely - some examine risk components and recurrence, others explore biological mechanisms, while yet others emphasize clinical management and therapeutic outcomes [20,22,23,25,26]. Differences between diagnostic factors and results make the comparison of the studies more difficult.

In addition, factors such as age, systemic medical conditions, inherited traits, and patient adherence to oral care are not systematically tested, which may influence results. Considering all parameters, gingival enlargement associated with orthodontic treatment is a multifactorial condition comprising microbiological alterations, mechanical factors, the overall duration of therapy, material-associated effects, and host-mediated responses. Overall, the information available indicates the necessity for a comprehensive approach consisting of stringent plaque control, frequent evaluation, and immediate action for both prevention and management of orthodontically induced gingival enlargement. Surgical techniques are essential in more complex conditions, while non-surgical methods are still preferred as the initial treatment. Future investigations should also focus on microbiome alterations, the various types of orthodontic appliances, and host-related factors for a better understanding of the involved mechanisms.

## Conclusions

Based on available information, gingival enlargement associated with orthodontic therapy is a multifactorial disease influenced by material-related, mechanical, and microbiological factors. Orthodontic devices may induce plaque accumulation, mechanical discomfort, and biological reactions at the gingiva, thereby leading to the emergence of gingival enlargement. Although non-surgical methods and enhanced dental hygiene can potentially minimize inflammatory responses, they are often inadequate for ultimate remission. In severe or chronic cases, surgery or laser technologies could be essential. The findings were synthesized narratively. From a clinical point of view, the prevention and treatment of such conditions may rely on prompt detection, effective biofilm control, and patient adherence. Management outcomes could be further enhanced through an approach that incorporates periodontal and orthodontic treatment. Nevertheless, a shortage of longitudinal evidence and a range of research designs restricts the current number of findings. To gain a greater awareness of the fundamental mechanisms and to strengthen prophylactic and therapeutic techniques for gingival enlargement associated with orthodontic appliances, a more meticulously organized longitudinal and interventional investigation is essential.

## References

[1] Mavrogiannis M, Ellis JS, Thomason JM, Seymour RA (2006). The management of drug-induced gingival overgrowth. J Clin Periodontol.

[2] Kantarci A, Augustin P, Firatli E, Sheff MC, Hasturk H, Graves DT, Trackman PC (2007). Apoptosis in gingival overgrowth tissues. J Dent Res.

[3] Marshall RI, Bartold PM (1998). Medication induced gingival overgrowth. Oral Dis.

[4] Di Spirito F, Amato A, Di Palo MP, Cannatà D, Giordano F, D'Ambrosio F, Martina S (2023). Periodontal management in periodontally healthy orthodontic patients with fixed appliances: an umbrella review of self-care instructions and evidence-based recommendations. Dent J (Basel).

[5] Naranjo AA, Triviño ML, Jaramillo A, Betancourth M, Botero JE (2006). Changes in the subgingival microbiota and periodontal parameters before and 3 months after bracket placement. Am J Orthod Dentofacial Orthop.

[6] Huser MC, Baehni PC, Lang R (1990). Effects of orthodontic bands on microbiologic and clinical parameters. Am J Orthod Dentofacial Orthop.

[7] Paolantonio M, Festa F, di Placido G, D’Attilio M, Catamo G, Piccolomini R (1999). Site-specific subgingival colonization by Actinobacillus actinomycetemcomitans in orthodontic patients. Am J Orthod Dentofacial Orthop.

[8] Sukontapatipark W, El-Agroudi MA, Selliseth NJ, Thunold K, Selvig KA (2001). Bacterial colonization associated with fixed orthodontic appliances. A scanning electron microscopy study. Eur J Orthod.

[9] Sallum EJ, Nouer DF, Klein MI, Gonçalves RB, Machion L, Sallum AW, Sallum EA (2004). Clinical and microbiologic changes after removal of orthodontic appliances. Am J Orthod Dentofacial Orthop.

[10] Karamani I, Kalimeri E, Seremidi K, Gkourtsogianni S, Kloukos D (2022). Chlorhexidine mouthwash for gingivitis control in orthodontic patients﻿: a systematic review and meta-analysis. Oral Health Prev Dent.

[11] Zanatta FB, Ardenghi TM, Antoniazzi RP, Pinto TM, Rösing CK (2014). Association between gingivitis and anterior gingival enlargement in subjects undergoing fixed orthodontic treatment. Dental Press J Orthod.

[12] Crego-Ruiz M, Jorba-García A (2023). Assessment of the periodontal health status and gingival recession during orthodontic treatment with clear aligners and fixed appliances: a systematic review and meta-analysis. Med Oral Patol Oral Cir Bucal.

[13] Atack NE, Sandy JR, Addy M (1996). Periodontal and microbiological changes associated with the placement of orthodontic appliances. A review. J Periodontol.

[14] Persson R, Svendsen J, Daubert K (1989). A longitudinal evaluation of periodontal therapy using the CPITN index. J Clin Periodontol.

[15] Gomes SC, Varela CC, da Veiga SL, Rösing CK, Oppermann RV (2007). Periodontal conditions in subjects following orthodontic therapy. A preliminary study. Eur J Orthod.

[16] Maboudi A, Fekrazad R, Shiva A, Salehabadi N, Moosazadeh M, Ehsani H, Yazdani O (2023). Gingivectomy with diode laser versus the conventional scalpel surgery and nonsurgical periodontal therapy in treatment of orthodontic treatment-induced gingival enlargement: a systematic review. Photobiomodul Photomed Laser Surg.

[17] Vincent-Bugnas S, Borsa L, Gruss A, Lupi L (2021). Prioritization of predisposing factors of gingival hyperplasia during orthodontic treatment: the role of amount of biofilm. BMC Oral Health.

[18] Simon CP, Motoc AG, Simon GA, Brezovan D, Muselin F, Cristina RT, Bratu DC (2020). Gingival proliferative growth - stress and cytoarchitecture related with fixed and mobile orthodontic therapy. Rom J Morphol Embryol.

[19] Rădeanu AC, Surpăţeanu M, Munteanu CM, Liliac IM, Popescu AD, Andrei EC, Pătru CL (2024). Periodontal changes induced by fixed orthodontic therapy. Med Pharm Rep.

[20] Jadhav T, Bhat KM, Bhat GS, Varghese JM (2013). Chronic inflammatory gingival enlargement associated with orthodontic therapy - a case report. J Dent Hyg.

[21] Gursoy UK, Sokucu O, Uitto VJ (2007). The role of nickel accumulation and epithelial cell proliferation in orthodontic treatment-induced gingival overgrowth. Eur J Orthod.

[22] Bellamine M, Ousehal L, Kissa J (2012). Orthodontic treatment and gingival hyperplasia: a case report. [Article in French]. Odontostomatol Trop.

[23] Barack D, Staffileno H, Sadowsky C (1985). Periodontal complication during orthodontic therapy: a case report. Am J Orthod.

[24] Farhadian N, Bidgoli M, Jafari F, Mahmoudzadeh M, Yaghobi M, Miresmaeili A (2015). Comparison of electric toothbrush, Persica and chlorhexidine mouthwashes on reduction of gingival enlargement in orthodontic patients: a randomised clinical trial. Oral Health Prev Dent.

[25] Convissar RA, Diamond LB, Fazekas CD (1996). Laser treatment of orthodontically induced gingival hyperplasia. Gen Dent.

[26] Gama SK, De Araújo TM, Pozza DH, Pinheiro AL (2007). Use of the CO2 laser on orthodontic patients suffering from gingival hyperplasia. Photomed Laser Surg.

[27] Al-Mutairi MA, Al-Salamah L, Nouri LA (2024). Microbial changes in the periodontal environment due to orthodontic appliances: a review. Cureus.

[28] Teodorescu IM, Preoteasa E, Preoteasa CT, Murariu-Măgureanu C, Teodorescu C (2025). Association of systemic pathologies on dental, periodontal and orthodontic status in children. Biomedicines.

[29] Stoyanova-Ivanova A, Georgiev V, Martins JN (2025). Nickel ion release in nickel-containing orthodontics archwires: a narrative review of in vitro and in vivo studies. Dent J (Basel).

[30] Amato A, Martina S, De Benedetto G, Michelotti A, Amato M, Di Spirito F (2025). Hypersensitivity in orthodontics: a systematic review of oral and extra-oral reactions. J Clin Med.

[31] Mester A, Onisor F, Mesaros AS (2022). Periodontal health in patients with self-ligating brackets: a systematic review of clinical studies. J Clin Med.

[32] Rossini G, Parrini S, Castroflorio T, Deregibus A, Debernardi CL (2015). Periodontal health during clear aligners treatment: a systematic review. Eur J Orthod.

